# Assessment of neonatal intensive care unit nurses’ compliance with standard precautions of infection control and identification of enabling factors

**DOI:** 10.1186/s43094-022-00456-y

**Published:** 2023-01-23

**Authors:** Dina K. Abou El Fadl, Yasmin A. F. Aly, Ebtissam Abdel Ghaffar Darweesh, Nagwa A. Sabri, Marwa Adel Ahmed

**Affiliations:** 1 Pharmacy Practice & Clinical Pharmacy Department, Faculty of Pharmacy, Future University in Egypt, Cairo, Egypt; 2 Paediatrics Department, Faculty of Medicine, Ain Shams University, Cairo, Egypt; 3 Clinical Pharmacy Department, Faculty of Pharmacy, Ain Shams University, Cairo, Egypt

**Keywords:** Neonatal intensive care unit, Infection control, Standard precautions, Nurses

## Abstract

**Background:**

Rigorous implementation of infection prevention and control practices by healthcare workers in different healthcare settings is of utmost importance. Neonates, particularly preterm babies in neonatal intensive care units, are a vulnerable population at high risk for developing nosocomial infections. Nurses have the greatest risk of spreading healthcare-associated infections among patients and healthcare workers. This study was conducted to assess the compliance of neonatal intensive care unit nurses with standard precautions of infection control and to identify the potential influencing factors.

**Results:**

This was a cross-sectional study, whereby the compliance of a total of 58 neonatal intensive care unit nurses with standard precautions of infection control was assessed using the Arabic version of the Compliance with Standard Precautions Scale (CSPS-A). Student’s t test, ANOVA test, and post hoc test were used for analysis.

A suboptimal compliance rate (66.7%) was detected, with the highest for disposal of sharp articles into sharps boxes (86.2%) and the lowest for disposal of sharps box not only when full (27.6%). Significant differences were observed when participants were grouped according to their clinical experience and qualifications, where participants with longer clinical experience displayed higher mean scores for the use of protective devices score (*P* = 0.024), disposal of sharps score (*P* = 0.003), and total CSPS score (*P* = 0.006).

**Conclusions:**

Clinical experience and educational qualifications are key factors that impact nurses’ compliance with infection control practices. Nurses should receive up-to-date evidence-based educational and practical sessions that link theory to clinical practice and elucidate the importance of accurate implementation of proper infection prevention and control practices.

## Background

Nosocomial infections in neonatal intensive care units (NICUs) have recently been identified as one of the major issues for NICU premature infants [1, 2]. Center for Disease Control and Prevention (CDC) describes all neonatal infections, acquired either at birth or during hospitalization, as nosocomial, unless there is evidence of transplacental transmission [3–5].

Neonates represent a distinctive and highly vulnerable patient population. Premature neonates are more susceptible to nosocomial infections due to their immature immune systems, low birth weights, low gestational ages, the use of intravascular catheters, the need for supporting devices, and prolonged hospitalization [6–8]. Therefore, the prevention of healthcare-associated infections must remain a priority for NICUs [9].

Over the past few decades, advanced medical technologies and therapeutic interventions have enhanced the survival and the quality of life for NICU neonates, especially those born with extreme prematurity or congenital defects. These do tend, however, to be associated with an increased risk of nosocomial infections due to exposure to multiple healthcare workers (HCWs) and the use of invasive technologies [10, 11].

Nosocomial infections are considered one of the leading causes of neonatal morbidity and mortality in NICUs with significant impact on the quality of care by increasing the duration and the cost of hospitalization [12–14]. The incidence of nosocomial infections NICUs s approximately 30%. More than one million neonatal deaths are estimated annually worldwide. In developing countries, nosocomial infections account for approximately 40% of reported neonatal deaths [15–17].

The neonatal intensive care unit is a unique setting, with aspects of intensive care, long-term care facility, and ward care mixed together. Despite the advanced protocols of infection prevention and control in different healthcare settings, including NICUs, ineffective implementation of infection control practices by HCWs appears to play a pivotal role in dissemination of nosocomial infections. Since nurses are the front-line healthcare practitioners who have the most consistent direct day-to-day contact with patients, they have the greatest risk of spreading healthcare-associated infections as well as cross-infections among patients and fellow healthcare practitioners [18, 19]. Infection prevention and control practices are thus critically vital to maintain provision of healthcare services while ensuring safety of patients and fellow HCWs [20].

With the ongoing coronavirus disease 2019 (COVID-19) pandemic disrupting most specialized healthcare services worldwide, NICUs are undergoing an intense rapid remodeling of the organization and quality of care, with special consideration to infection prevention and control measures [21].

The WHO identified Standard Precautions (SPs) as a major part of the basic set of infection control and prevention guidelines [22]. SPs represent the minimum level of precautions that should be adopted by HCWs when providing patient’s care [23, 24]. They comprise the basic infection control practices that provide a high level of protection to patients, HCWs and visitors [25]. Previous studies have demonstrated the importance of strict compliance of HCWs with SPs to reduce the incidence of nosocomial infections in NICUs and other healthcare settings [23].

Accordingly, the compliance of HCWs, particularly nurses, should be regularly assessed using a valid and reliable assessment tool. Hence, the Compliance with Standard Precautions Scale (CSPS) was developed in 2010 in consonance with the guidelines of the Standard Precautions of the World Health Organization.

The CSPS was comprehensively designed to assess the nurses’ self-reported compliance with the main SPs dimensions and to describe their daily routine in applying infection control practices in their work [26].

The aim of the presented study was thus to assess NICU nurses’ compliance with SPs of infection control and to identify the influencing factors.

## Methods

### Study design

A descriptive cross-sectional study.

### Setting and participants

The study was conducted in the NICU of Ain Shams University Hospitals in Cairo, Egypt. The total population of nurses was 72. A convenient sample of 58 nurses agreed to participate in the study.

### Ethics approval

Prior to research conduction, approval for using the CSPS-A was received from the CSPS developer and copyright holder (A030D12-201809). The approval for both the scientific and ethical aspects of the study was obtained from the Committee of Ethics of Faculty of Pharmacy, Ain Shams University (*serial number of protocol: Ph.D. No.73*) and the Joint Committee for the Protection of Human Subjects in Research of the Health Science Centre, Faculty of Pharmaceutical Sciences & Pharmaceutical Industries, Future University in Egypt (*serial number of protocol: REC-FPSPI-13/97*).

The clinical pharmacist, as the primary investigator, explained the purpose of the study and the average time needed for filling the questionnaire to the participants and informed them that participation was totally voluntary and that they had the right to withdraw from the study at any time without any obligations. No incentives were given to the participants.

Protection of the participants’ anonymity and confidentiality was assured, and they were instructed not to write their names or any personal details that could reveal their identity in any part of the questionnaire.

### Study instrument

The Arabic version of a two-part self-report questionnaire was used. The first part included questions on participants’ demographic data such as age, gender, marital status, level of education, clinical exposure and infection control workshops’ attendance. The second part included the 20-item Compliance with Standard Precautions Scale (CSPS) [23].

The development of CSPS was followed by a comprehensive psychometric testing conducted on a sample of 453 participants, including nursing staff and students. Findings revealed that CSPS had satisfactory reliability, construct validity as well as concurrent validity. Moreover, CSPS went through a cross-cultural pilot test that included 19 experts from 16 different countries. The test results revealed that CSPS is relevant and applicable to most developed and developing regions [27].

CSPS has been translated to several languages, including Arabic, Korean, Mainland Chinese, Greek, Italian, Spanish, Portuguese, and Turkish [28]. Since Arabic is the native language of the Egyptian nurses, the questionnaire used in this study was the Arabic version of the CSPS (CSPS-A). After cultural adaptation of the original CSPS to Arabic language, psychometric testing was performed in a study that included a sample of Saudi nursing students and revealed good internal consistency and stability reliability along with acceptable content and construct validity [23].

The CSPS items address issues related to daily clinical practice at healthcare settings. This includes personal protective equipment (PPE) use (item 7, 10, 13, 14, 15, and 16), decontamination of spills and used articles (item 18, 19, and 20) disposal of sharp objects (item 4, 5, and 6), disposal of biological wastes (item 17) and prevention of cross-infection (item 1, 2, 3, 8, 9, 11, and 12). The scale includes 20 items: 16 positively worded items and 4 negatively worded items (item 2, 4, 6, and 15).

The response format is a 4-point adjectival scale, from never (0), seldom (1), sometimes (2) to always (3). For score calculation only the “always” option in positively worded items and the “never” option in negatively worded items are given a score of 1, while the other options are given a score of zero. The total scores range from 0 to 20, and a higher score indicates a better compliance with standard precautions [27].

The total compliance rate refers to the average percentage of compliance with all 20 items. In general, it is optimal when compliance rate is > 90%, satisfactory between 80 and 89%, suboptimal between 50 and 79%, and poor for < 49%. The item compliance rate refers to the mean score of each item [29].

### Data collection

The paper printed questionnaire was introduced to the nurses so that data could be collected in the first hour of their working shifts in the NICU. This was carried out from May to June 2019. The participants received instructions on how to complete the questionnaire before they were handed it. As the nurses filled the questionnaire, the primary investigator was available to answer any question. The completion of the questionnaire took approximately 10–15 min.

### Data management and analysis

The collected data were revised, coded, tabulated and introduced to a PC using Statistical Package for Social Science (IBM Corp. Released 2011. IBM SPSS Statistics for Windows, Version 20.0. Armonk, NY: IBM Corp). Data were presented and suitable analysis was done according to the type of data obtained for each parameter. For the descriptive statistics, data were tested for normality with Kolmogorov–Smirnov test and expressed as mean (standard deviation) for normally distributed data or median (interquartile range) for non-normally distributed numerical data. The frequency and percentage of non-numerical data were calculated.

Regarding the analytical statistics, Student’s *t* test was used to assess the statistical significance of the difference between two study group means, ANOVA test was used to assess the statistical significance of the difference between more than two study group means, and Bonferroni post hoc test was used for comparisons between all possible pairs of group means.

### Sample size justification

Sample size was calculated setting the type 1 error (*α*) at 0.05 and the confidence interval width at 0.1 (margin of error 5%). Result from previous study showed that Concerning the level of nurses’ practices regarding infection prevention measures, 4.7% of the Yemeni nurses had a poor level of practices (< 50%) [30]. Given that the total nurses’ population in our study site is 72 nurses, the calculated needed sample size is 36 nurses. However, we included 58 nurses to compensate for dropout rate and incomplete and non-reliable questionnaires.

## Results

### Socio-demographic characteristics of participants

The median age of the participants was 24 years old, so the age cutoff was set at 24.

The mean age of participants was 25.1 (± 5.5) years, and the mean duration of clinical experience was 4.9 (± 5.3) years, with a median of 3 years. Females represented 60.3% of the participants. Single nurses represented 72.4% of the participants, 63.8% were graduated from the Technical Institute of Nursing, and about 78% reported attending infection control courses/workshops (Table 1).

**Table 1:** Description of participants’ socio-demographic characteristics

	Mean	± SD	Minimum	Maximum	Median (IQR)*
Age (Years)	25.19	5.51	18	45	
Clinical experience (Years)	4.92	5.37	0.5	24	3 (2–5)

		*N*	%
Age-group (Years)	< 24 Years	28	48.3
	≥ 24 Years	30	51.7
Gender	Male	23	39.7
	Female	35	60.3
Marital status	Single	42	72.4
	Married	12	20.7
	Divorced	4	6.9
Clinical experience(Years)	< 3 Years	27	46.6
	≥ 3 Years	31	53.4
Qualifications	Nursing BSc	14	24.1
	Nursing Diploma	7	12.1
	Technical Institute	37	63.8
Attending Infection Control Courses	No	13	22.4
	Yes	45	77.6

*Interquartile range

### Compliance rates of participants in different CSPS items

The overall compliance rate among the participants was found to be suboptimal (66.7%) (Table 2).

**Table 2:** Participants’ compliance rates in CSPS items

Use of protective devices (PPD)	Compliance rate (%)	Mean score	± SD
	68.2	4.09	1.45
Removing personal protective equipment (PPE) in a designated area	70.7		
Wearing gloves	84.5		
Wearing surgical mask alone or with goggles and face shield	48.3		
Covering mouth and nose with mask	75.9		
Not Reusing masks or disposable PPE	69.0		
Wearing gown or apron	60.3		

Disposal of sharps	Compliance rate (%)	Mean score	± SD
	54	1.62	0.89
Not Recapping used needles	48.3		
Putting sharp articles into sharps boxes	86.2		
Disposal of sharps box not only when full	27.6		

Disposal of wastes	Compliance rate (%)	Mean score	± SD
	79	0.79	0.41
Placing contaminated wastes in red plastic bags	79		

Decontamination of spills and used articles	Compliance rate (%)	Mean score	± SD
	74	2.22	1.06
Decontaminating surfaces and equipment after use	75.9		
Wearing gloves during decontamination	65.5		
Spillage clean up	81.0		

Prevention of cross-infection	Compliance rate (%)	Mean score	± SD
	66	4.62	1.53
Washing hands between patient contacts	82.8		
Not using water only for hand washing	67.2		
Using alcohol hand rubs as an alternative	37.9		
Taking a shower in case of extensive splashing	56.9		
Covering wounds or lesions	56.9		
Changing gloves between patients	79.3		
Hands decontamination after gloves removal	81.0		
Overall compliance rate	66.7		

**Fig. 1:**
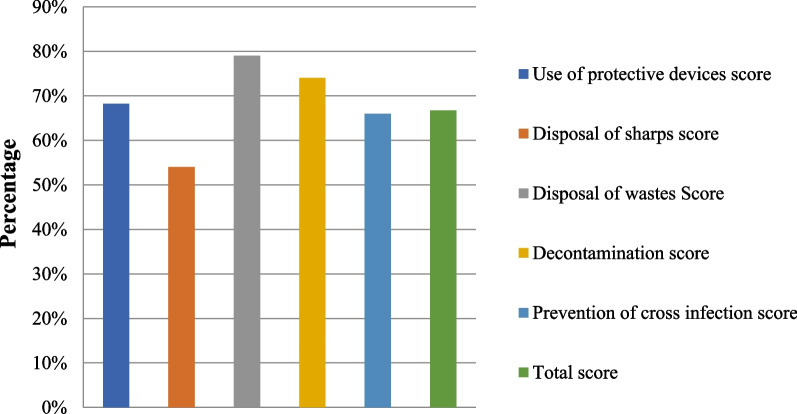
Participants' average compliance rates in CSPS dimensions and total CSPS score

The CSPS dimension with the highest average compliance rate was the disposal of wastes (79%) and that with the lowest average compliance rate was the disposal of sharps (54%) (Fig. 1). Among the 20 items of the CSPS scale, the highest compliance rate was reported for putting sharp articles into sharps boxes (86.2%), whereas the lowest compliance rate was reported for the disposal of sharps box not only when full (27.6%).

### Comparison of participants’ CSPS scores based on their demographic characteristics

**Fig. 2:**
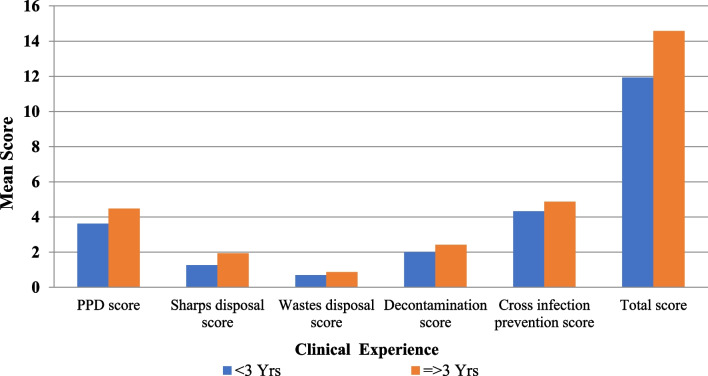
Comparison of CSPS dimension scores and total CSPS score between participants regarding their clinical experience years. PPD: Personal protective devices

**Fig. 3:**
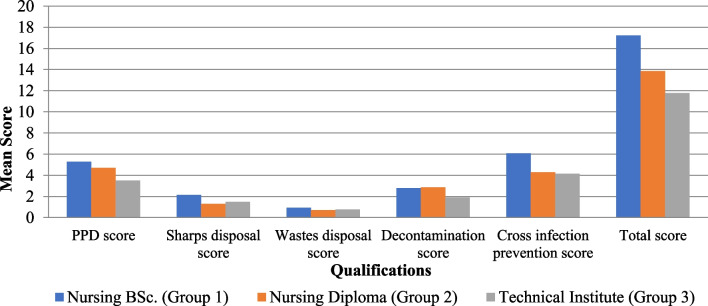
Comparison of CSPS dimension scores and total CSPS score between participants regarding their qualifications. PPD: Personal protective devices

- There were no significant differences in the mean CSPS dimensions scores and the total CSPS scores between the participants based on their age-group (*p* = 0.103), gender (*p* = 0. 434), marital status (*p* = 0.66), and whether or not they attended additional infection control courses (*p* = 0.96) (Table 3).
- There was a significant difference between participants with different clinical experience durations in the mean PPD score (*P* = 0.024), disposal of sharps score (*P* = 0.003), and total CSPS score (*P* = 0.006), with higher mean scores among participants with longer clinical experience (Fig. 2).
- There was a significant difference between participants with different qualifications in the mean PPD score (*p* = *0.001)*, disposal of sharps score (*p* = *0.03)*, decontamination score (*p* = *0.005)*, prevention of cross-infection score (*p* = *0.001)*, and the total CSPS score (*p* = *0.001)*, with higher mean scores among participants holding nursing B.Sc. (Fig. 3).

**Table 3:** Comparison of CSPS scores between participants regarding their different socio-demographic characteristics

	Age-group				p*
	< 24		≥ 24		
	Mean	± SD	Mean	± SD	
Use of protective devices (PPD) score	3.75	1.51	4.40	1.35	0.089
Disposal of sharps score	1.46	0.84	1.77	0.94	0.201
Disposal of wastes score	0.79	0.42	0.80	0.41	0.896
Decontamination score	2.00	1.12	2.43	0.97	0.121
Prevention of cross-infection score	4.50	1.80	4.73	1.26	0.572
Total CSPS score	12.50	4.23	14.13	3.13	0.103

	Gender				p*
	Male		Female		
	Mean	± SD	Mean	± SD	
Use of protective devices (PPD) score	4.00	1.28	4.14	1.57	0.71
Disposal of sharps score	1.96	0.93	1.40	0.81	0.019
Disposal of wastes score	0.87	0.34	0.74	0.44	0.227
Decontamination score	2.17	0.98	2.26	1.12	0.773
Prevention of cross-infection score	4.83	1.67	4.49	1.44	0.412
Total CSPS score	13.83	3.39	13.03	4.00	0.434

	Marital status						p**
	Single		Married		Divorced		
	Mean	± SD	Mean	± SD	Mean	± SD	
Use of protective devices (PPD) score	4.05	1.43	4.17	1.70	4.25	1.26	0.94
Disposal of sharps score	1.52	0.86	1.75	0.97	2.25	0.96	0.26
Disposal of wastes score	0.79	0.42	0.75	0.45	1.00	0.00	0.56
Decontamination score	2.19	1.06	2.25	1.14	2.50	1.00	0.85
Prevention of cross-infection score	4.64	1.64	4.42	1.08	5.00	1.83	0.79
Total CSPS score	13.19	3.89	13.33	3.80	15.00	2.16	0.66

	Clinical experience				p*
	< 3 Yrs		≥ 3 Yrs		
	Mean	± SD	Mean	± SD	
Use of protective devices (PPD) score	3.63	1.52	4.48	1.29	0.024
Disposal of sharps score	1.26	0.86	1.94	0.81	0.003
Disposal of wastes score	0.70	0.47	0.87	0.34	0.130
Decontamination score	2.00	1.18	2.42	0.92	0.134
Prevention of cross-infection score	4.33	1.69	4.87	1.36	0.185
Total CSPS score	11.93	4.15	14.58	2.92	0.006

	Qualifications						p**
	Nursing B.Sc.		Nursing Diploma		Technical Institute		
	Mean	± SD	Mean	± SD	Mean	± SD	
Use of protective devices (PPD) score	5.29	0.61	4.71	1.38	3.51	1.39	0.001^a^
Disposal of sharps score	2.14	0.86	1.29	0.76	1.49	0.87	0.03^b^
Disposal of wastes score	0.93	0.27	0.71	0.49	0.76	0.43	0.358
Decontamination score	2.79	0.43	2.86	0.38	1.89	1.17	0.005
Prevention of cross-infection score	6.07	0.62	4.29	1.11	4.14	1.51	0.001^c^
Total CSPS score	17.21	0.97	13.86	3.13	11.78	3.47	0.001^d^

	Attending infection control courses				p*
	No		Yes		
	Mean	± SD	Mean	± SD	
Use of protective devices (PPD) score	3.85	1.14	4.16	1.54	0.50
Disposal of sharps score	1.38	0.87	1.69	0.90	0.28
Disposal of wastes score	0.77	0.44	0.80	0.40	0.81
Decontamination score	2.31	1.03	2.20	1.08	0.75
Prevention of cross-infection score	5.00	1.47	4.51	1.55	0.31
Total CSPS score	13.31	3.50	13.36	3.87	0.96

*CSPS* Compliance with Standard Precautions Scale, *PPD* Personal protective devices

*Student’s *t* test; **ANOVA test, Bonferroni post hoc test

^a^Group 1 Vs. Group 2 nonsignificant (NS), Group 1 Vs. Group 3 significant (S) Group 2 Vs. Group 3 (S)

^b^Group 1 Vs. Group 2 (NS), Group 1 Vs. Group 3 (S) Group 2 Vs. Group 3 (S)

^c^Group 1 Vs. Group 2 (S), Group 1 Vs. Group 3 (S) Group 2 Vs. Group 3 (NS)

^d^Group 1 Vs. Group 2 (S), Group 1 Vs. Group 3 (S) Group 2 Vs. Group 3 (NS)

## Discussion

In the present study, the Arabic version of the Compliance with Standard Precautions Scale (CSPS-A) was used to assess compliance of NICU nurses with standard precautions of infection control.

The study results showed a suboptimal overall compliance rate among the study participants (66.7%). When compared to similar studies using the same tool, the compliance rate was found to be higher than that reported among nursing students in Saudi Arabia (61%) and among nursing students (53.5%) and nursing staffs (57.4%) in Hong Kong. Conversely, the compliance rate was lower than that reported among nursing staffs in Brazil (69.4%) [18, 27, 31].

This study revealed variability in the compliance rates of the participants in different CSPS items. Regarding the Disposal of Sharps**,** a high compliance rate was reported for the disposal of used sharp articles into sharps-only boxes (86.2%), whereas a low compliance rate was reported for not recapping used needles (48.3%) and for disposal of sharps box not only when full (27.6%).

Similarly, this erroneous practice by nursing students and staffs has been reported in previous studies [32–34]. A study among nursing students in Saudi Arabia showed a high compliance rate for the disposal of used sharp articles into sharps-only boxes (84.3%) and a low compliance rate for not recapping used needles (49.2%) [18]. A comparable study in Hong Kong revealed a high compliance rate for the disposal of used needles in a sharps-only box (95.3%) and a low compliance rate for not recapping used needles (49.3%) [35]. A Turkish study among Turkish nursing and midwifery students revealed a high compliance rate for both the disposal of used sharp articles into sharps-only boxes (86.5%) and not recapping used needles (89.4%)[36].

Recapping used needles is considered a prime risk for needlestick injury; accordingly it is highly important to dispose used needles immediately in sharps-only boxes [36]. Used needles should be recapped only if this is required in a certain procedure or if sharps-only boxes are not available, and even in such circumstances recapping should be done either by the one-handed technique or by using a mechanical device [37].

Regarding the CSPS items of cross-infection prevention, a high compliance rate was reported for washing hands between patients’ contacts (82.8%), immediately decontaminating hands after removing gloves (81%) and for changing gloves after contact with each patient (79.3%). These results are comparable to those reported in previous studies in Hong Kong, Jordan, Turkey, and Saudi Arabia [18, 34, 35, 38].

An earlier study denoted attending training infection control programs or workshops as a predictor of hand hygiene practice among nursing students [39]. This could justify the findings of this study where a high percentage of participants reported attending infection control courses and workshops (77.6%).

Conversely, the compliance rate for the item of wearing a surgical mask alone or along with goggles and face shield was low (48.3%) and this may be attributed to the availability of these protective equipment in the NICU. This goes in line with the results of a former Turkish study, where a low compliance rate was reported for wearing personal protective equipment, such as masks, goggles or gowns whenever there was a possibility of blood or other body fluids splashing on clothes, due to the lack of such equipment in clinical settings [38]. According to Colet et al. one plausible explanation for the high compliance rate in wearing a surgical mask alone or along with goggles and face shield (72.9%) is the accessibility of these equipment in clinical settings [18].

Regarding wastes disposal, the participants reported a high compliance rate. Optimally, healthcare waste disposal should never be mixed; wastes contaminated with blood or any body fluids should be placed in red plastic bags. These findings were different from those revealed by similar previous studies where low compliance rates were reported for placing contaminated wastes in red plastic bags [18, 40]. One qualitative study attributed improper waste disposal to lack of awareness and inadequate training on healthcare waste disposal [41].

On studying other potential factors affecting the nurses’ compliance with SPs, it was found that participants holding Bachelor’s degree in nursing as well as those who had three or more years of clinical experience reported higher compliance rates. This agrees with the findings of previous studies among nursing students, where senior students exhibited higher compliance rates with SPs of infection control [18, 38]. This could be related to the fact that nursing students in the latter years of the nursing programs have greater exposure to clinical settings and thus more clinical experience, which might enhance their implementation of SPs.

Findings of the present study suggest that clinical experience duration is a substantial factor affecting the nurses’ compliance with SPs. Nurses with longer durations of clinical experience become more acquainted with the infection control guidelines and protocols. The findings are also consonant with Patricia Benner’s theory “From Novice to Expert.” According to this theory, expert nurses develop skills and understanding of patient care over time through a sound educational base as well as a multitude of experiences.

The theory described five different levels of nursing experience; novice, advanced beginner, competent, proficient, and expert [42]. Direct experience has been described as an indispensable tool in the elaboration and enhancement of psychomotor and decision making skills [43].

Addressing infection prevention and control can be challenging especially in complex healthcare settings as NICUs. One of the challenges is the increased number of diverse interprofessional practices within these settings and subsequently higher risk of infection. In order for HCWs to practice safely, their knowledge and skills should be up-to-date and evidence-based. Thus, healthcare organizations should provide periodic effective educational and training programs that link theory to clinical practice [44].

Infection prevention and control is also considered one of the primary goals of antimicrobial stewardship programs. Antimicrobial stewardship involves more than antibiotic prescribing alone; effective adherence of HCWs and patients to SPs will prevent infection transmission and thus remove the need for antibiotics. The role of antimicrobial stewardship is thus to prevent infection in the first place, and one challenge for healthcare organizations is to provide appropriate training, education and support for HCWs so that antimicrobial stewardship is embedded within their role [19].

Given the current global crisis of COVID-19 pandemic, healthcare systems should also ponder innovative ways to support and enrich their infection control programs. Antimicrobial stewardship efforts may also be redirected to help with COVID-19 combat efforts. These efforts should include investing in information technology and personnel. Beyond the current pandemic, substantial resources should be invested to enhance and sustain infection control infrastructure at the local, regional and national levels. Besides, new investment in training and expanding the infection control workforce will be crucial [45].

Infectious disease clinical pharmacist is one of the core members of antimicrobial stewardship team as well as the infection control committee at healthcare facilities with a prominent educational and vocational role aimed at patients and HCWs at the point of care [46].

This includes providing robust up-to-date evidence-based educational and training programs that link theory to clinical practice and elucidate the importance of accurate implementation of proper infection prevention and control practices while focusing on the points of weakness revealed by the results of the periodic assessment of compliance with SPs.

Our study addresses a timely and important problem regarding infection control in clinical settings. Considerable findings are revealed regarding the compliance of NICU nurses with standard precautions of infection control; however, there are some limitations. Using a self-report tool may have allowed some response bias such as social desirability and acquiescence. Also, the fact that the study was conducted in a single NICU, the use of a convenience sampling technique and the small sample size hinder the generalizability of the results. Hence, wider settings and larger sample sizes are recommended for future studies.

It also is worth noting that the current study design initially included further assessment of the impact of clinical pharmacist-led education and training programs on enhancing the adherence of NICU nurses to infection control measures; however, the mitigation strategies adopted in NICU to combat the COVID-19 pandemic and the restricted access to the unit hindered the implementation of the program. Nevertheless, the program is currently in the preparation phase with the aid of technologies and online tools that may help to overcome the barrier of limited access and thus allow prompt implementation.

## Conclusions

Within this study limitation, it could be concluded that clinical experience and educational qualifications are key factors that impact nurses’ compliance with standard precautions of infection control. Findings of the study emphasize the need of implementation of constant supervision on the quality of care in NICU as well as the necessity to train nurses to this specific work. It is therefore imperative to provide periodic up-to-date education and training programs for nurses in NICUs and other healthcare settings.

## Data Availability

The datasets used during the current study are available from the corresponding author upon reasonable request.

## Appendix

### Compliance with standard precautions scale (CSPS)

Please mark a ✔ in the box that best reflects your current clinical practice.

Please answer all 20 questions.

Approval for using the CSPS-A was received from the CSPS developer & copyright holder (A030D12-201809).

## Abbreviations

NICUs: Neonatal intensive care units
CDC: Center for Disease Control and Prevention
HCWs: Healthcare workers
COVID-19: Coronavirus disease 2019
SPs: Standard precautions
CSPS: Compliance with Standard Precautions Scale
PPE: Personal protective equipment
